# Age-Dependent Differences in Exercise Response Among Healthy Women: Impact on Inflammation, Lipids Profile and Glucose

**DOI:** 10.3390/biomedicines14030575

**Published:** 2026-03-04

**Authors:** Shamma Almuraikhy, Maha Sellami, Monoem Haddad, Najeha Rizwana Anwardeen, Mariam Al-Mohannadi, Mohamed A. Elrayess

**Affiliations:** 1 Biomedical Research Center, QU Health, Qatar University, Doha 2713, Qatar; salmuraikhy@qu.edu.qa (S.A.); n.anwardeen@qu.edu.qa (N.R.A.); 2 Sport Coaching Department, College of Sport Sciences, Qatar University, Doha 2713, Qatar; msellami@qu.edu.qa (M.S.);; 3 College of Sport Sciences, Qatar University, Doha 2713, Qatar; mhaddad@qu.edu.qa; 4 College of Medicine, QU Health, Qatar University, Doha 2713, Qatar

**Keywords:** physical activity, age interaction, women’s health, fasting blood sugar (FBS), tumor necrosis factor alpha (TNF α), lipid profile, fat-free mass, oxidative stress, telomere length, mixed effects models

## Abstract

**Background:** Inflammatory and metabolic risk factors are associated with adverse health outcomes among aging women. Physical activity may reduce these detrimental changes, helping to promote healthier aging. **Methods:** Seventy-nine non-obese women, aged 20–50 years, completed a supervised 4–8 week aerobic training program with measurements obtained before and after the intervention. The 20–30-year group (*n* = 29) completed a 4-week training program, with 13 participants fasting during training, while the 30–50-year group (*n* = 50) completed an 8-week program. Fasting blood sugar (FBS), lipid profile, insulin, Homeostatic Model Assessment for Insulin Resistance (HOMA IR), body composition, multiple cytokines, oxidative stress markers and leukocyte telomere length were assessed. Mixed-effects linear models were used to test age-by-activity (before versus after) interactions, adjusting for body mass index (BMI), fasting status and training duration. **Results:** Physical activity was associated with a higher superoxide dismutase (SOD) activity, lower tumor necrosis factor alpha (TNF α) concentrations, increased weekly Metabolic Equivalent of Task (METs) and a modest reduction in high-density lipoprotein (HDL) cholesterol. Significant age-by-activity interactions were identified for fat-free mass, total cholesterol, HDL cholesterol, FBS and TNF α, exhibiting attenuated or reversed age-related slopes for these traits after training. Specifically, older active women exhibited less age-related increases in FBS and TNF α and greater age-related reductions in total cholesterol, whereas the preservation of fat-free mass was more pronounced among younger participants. **Conclusions:** A short moderate-intensity aerobic program was sufficient to improve antioxidant defenses and inflammatory status and reshape age-group-specific responses to the training of selected glycemic, lipid, inflammatory and functional markers in healthy women, partly mitigating adverse age-associated changes, particularly in older participants. By modeling age-by-activity interactions across various metabolic and inflammatory risk factors, this study provides evidence that short-term moderate aerobic training can reshape age-group-specific cardiometabolic responses to training.

## 1. Introduction

Aging is a fundamental biological process that contributes to various health problems, including metabolic disease, chronic inflammation, increased adiposity, insulin resistance, and hormonal changes, all of which accelerate health decline and the aging process. Dysfunctional adipose tissue increases visceral fat accumulation and low-grade inflammation, driving insulin resistance. Simultaneously, reduced physical activity in later life exacerbates muscle loss, fat deposition, and diminished functional fitness, compounding these adverse effects [1,2,3,4].

Midlife represents a critical window for the development of metabolic risk factors in women. Age-related increases in inflammation and metabolic dysfunction become increasingly pronounced from early adulthood, particularly among women aged 30–50 years. This life stage is characterized by hormonal fluctuations, shifts in body composition, and transition into perimenopause, all contributing to an increased risk of metabolic syndrome, central obesity, impaired glucose tolerance, and dyslipidemia. Epidemiological studies have shown that the prevalence of metabolic syndrome can exceed 30% among urban women in this age group, with abdominal obesity, elevated triglycerides, low HDL-C, and hypertension being the most common risk factors [5,6].

Physical activity is well established as a key intervention to counteract aging, mainly by reducing inflammation, lowering cholesterol, and improving insulin sensitivity and glucose metabolism. Aerobic and resistance exercise regimens in adult women have shown marked reductions in systemic cytokines, including interleukin-6 (IL-6) and C-reactive protein (CRP), improved lipid profiles, and enhanced metabolic control, thus mitigating multiple age-related hallmarks. Structured exercise increases muscle mass, lowers fat mass, and improves cardiovascular status, delaying the onset of health complications commonly seen in older women [7,8,9,10]. Recent findings confirm that continuous physical activity leads to improved insulin sensitivity, decreased inflammatory status, healthier lipid profiles, and slowed biological aging in women across adulthood. Therefore, promoting and maintaining adequate physical activity is essential for optimizing health and reducing age-related risks, especially for women entering and progressing through midlife [7,9,11,12,13]. However, the heterogeneity in exercise response with age indicates that individual factors such as baseline health status, comorbidities, and lifestyle can substantially influence the magnitude of functional and physiological gains [14].

Indeed, women aged 20 to 30 years typically exhibit healthier metabolic and inflammatory profiles, as well as better exercise responsiveness than older women. In contrast, midlife women experience more pronounced declines in metabolic health and increased inflammation if not actively mitigated. This highlights the critical window for preventive and therapeutic exercise strategies [7,15]. This study aimed to investigate how physical activity differentially impacts metabolic and inflammatory health markers in younger and older women, with a particular focus on identifying age-specific patterns of benefit and vulnerability.

## 2. Materials and Methods

### 2.1. Study Participants

Seventy-nine lean/overweight, apparently healthy women aged 20–50 years participated in this study. Inclusion criteria included a BMI of 20–30 kg/m^2^ and the absence of cardiovascular conditions, type 2 diabetes, muscle degeneration, blood clots, or neurological disorders clinically diagnosed via medical history and physical examination. Exclusion criteria included women younger than 20 or older than 50 years old and women with a BMI less than 20 or greater than 30 Kg/m^2^, an inability to participate in walking and running exercises, breathing and pulmonary disease, undergoing primary surgery, distant metastatic disease, or a previous or concomitant malignancy that would interfere with this treatment protocol and pregnancy. Participants following a specific diet regimen, untoward physical or physiological responses to any testing parameter throughout the study and a failure to complete all sessions were also excluded. All participants provided written informed consent prior to participation. The study protocols were approved by Qatar University Institutional Research Board (QU-IRB 1798-EA/23) in compliance with the regulations of the Qatar Ministry of Public Health (MoPH). The cohort was divided into two groups. The first group (*n* = 29), aged 20–30 years, completed a 4-week training program; among them, 13 participants were fasting during training. The second group (*n* = 50), aged 30–50 years, completed an 8-week training program. Most participants were premenopausal according to self-report; however, menopausal status was not systematically documented for all women and could not be included as a covariate in the analyses.

### 2.2. Training Session

Participants underwent 4- or 8-week aerobic training protocols aligned with ACSM and AHA guidelines [16,17,18,19,20]. The exercise program consisted of aerobic training performed at progressive intensities, beginning at 40–60% of HRmax and approximately 50% of VO_2_ peak, and increasing to 60–70% by the eighth week. All participants trained three times per week for 50 min per session. Metabolic Equivalent of Task (MET) values were calculated using data from the International Physical Activity Questionnaire (IPAQ) to quantify daily physical activity. METs were used to standardize exercise intensity and energy expenditure across individuals, allowing comparisons independent of body weight.

### 2.3. Clinical Parameters and Cytokines Measurements

Fasting blood samples were collected before and after exercise intervention. The clinical chemistry analyzer Mindray BS240 was used to measure fasting blood sugar, total cholesterol, triglycerides, HDL and LDL according to the manufacturer’s instructions. An insulin ELISA kit (Mercodia, London, UK) was used to measure insulin levels in serum samples according to the manufacturer’s instructions [21]. Insulin resistance was assessed using the Homeostatic Model Assessment of insulin resistance (HOMA-IR) using the following formula: HOMA = Fasting blood glucose (mmol/L) × Fasting insulin (mIU/mL)/22.5 [22]. A TANITA body composition monitor was used to measure body fat, fat-free mass, fat mass, and muscle mass [23]. Cytokines, including IL-1β, IL-1RA, IL-6, IL-8/ CXCL8, MCP-1/CCL2 and TNF-α and IL-10, were measured using Custom Premix Human Cyto Panel (HCYTA-60K-08C, Millipore, Burlington, MA, USA) using Luminex™ FLEXMAP 3D, according to the manufacturer’s instructions [24]. Standard curves were generated using Xponent software, and cytokine concentrations were subsequently quantified. Colorimetric activity assays (EIACATC and EIASODC) were used to measure the activities of superoxide dismutase and catalase, according to the manufacturer’s instructions (ThermoFisher Scientific, Waltham, MA, USA) [25].

### 2.4. Measurement of Telomere Length

PureLink^®^ Genomic DNA Kits (Invitrogen, Life Technologies, Carlsbad, CA, USA) were used for the isolation of genomic DNA from the clotted blood at the bottom of the serum tubes, according to the manufacturer’s instructions as described previously [26]. Telomere length was measured using Absolute Human Telomere Length Quantification qPCR Assay Kit (ScienCell, Carlsbad, CA, USA) according to the manufacturer’s instructions. Briefly, two qPCR reactions were prepared for each genomic DNA sample: one with telomere (TL) and one with single copy reference (SCR) primer stock solutions. qPCR reactions were prepared by adding a genomic DNA template (5 ng/µL) to the primer stock solution (TL or SCR) and GoldNStart TaqGreen qPCR master mix. qPCR was run using an initial denaturation of 95 °C for 10 min, followed by 32 cycles of denaturation at 95 °C for 20 s, annealing at 52 °C for 20 s, and extension at 72 °C for 45 s using StepOne™ Real-Time PCR System (ThermoFisher). For the quantification of TL, ∆Cq (TL) was quantified by assessing the TL cycle number difference between the two genomic DNA samples (sample of interest and the reference genomic DNA sample with known telomere length). For SCR, ∆Cq (SCR) was assessed by quantifying the SCR cycle number difference between the two genomic DNA samples (sample of interest and the reference genomic DNA sample with known telomere length). ∆∆Cq was calculated as ∆Cq (TL) − ∆Cq (SCR). Fold change was assessed as 2 − ∆∆Cq, and the TL was expressed as a T/S ratio.

### 2.5. Statistical Method

We examined the interaction between age and changes in the levels of various clinical/metabolic traits (cytokines, oxidative stress markers, lipid profile, and body composition markers) with physical activity using linear mixed-effects models fitted with lme4 R package. For each variable, we modelled trait values as a function of activity (before/after), age, and their interaction (age × activity), adjusting for BMI, fasting status, and activity duration, with a subject-specific random intercept to account for repeated measures. Estimated marginal age slopes within each activity condition were obtained using emtrends from the emmeans package, and the difference in age slopes (after–before) was tested using reverse pairwise contrasts. Significant interactions were visualized using scatterplots with fitted linear trends and 95% confidence intervals stratified by activity status, with corresponding *p*-values annotated on each plot. All analyses and visualizations were performed in R (version 4.2.1).

## 3. Results

### 3.1. General Characteristics of Participants

In this study as shown in Table 1, the pre training reflects participants before the exercise intervention, whereas the post training represents the same participants after completing the program. The exercise intervention was associated with higher superoxide dismutase levels (0.76 to 0.94 μ/mL; *p* = 0.001), lower TNF-α concentrations (11.18 to 6.64 pg/mL; *p* = 0.002), a modest reduction in HDL cholesterol (1.51 to 1.39 mmol/L; *p* = 0.019), and an increase in total weekly METs (1155 to 1794; *p* = 0.004), indicating a clear shift toward a more active physiological profile. Interestingly, there was also a trend toward improved insulin sensitivity, with HOMA-IR decreasing from 2.68 to 2.21, although this change did not reach statistical significance (*p* = 0.072), in line with prior evidence that exercise can enhance insulin sensitivity even when effects on HOMA-IR are modest or variable across studies (Table 1).

### 3.2. Age and Physical Activity Jointly Shape Metabolic and Physiological Traits

**Figure 1:**
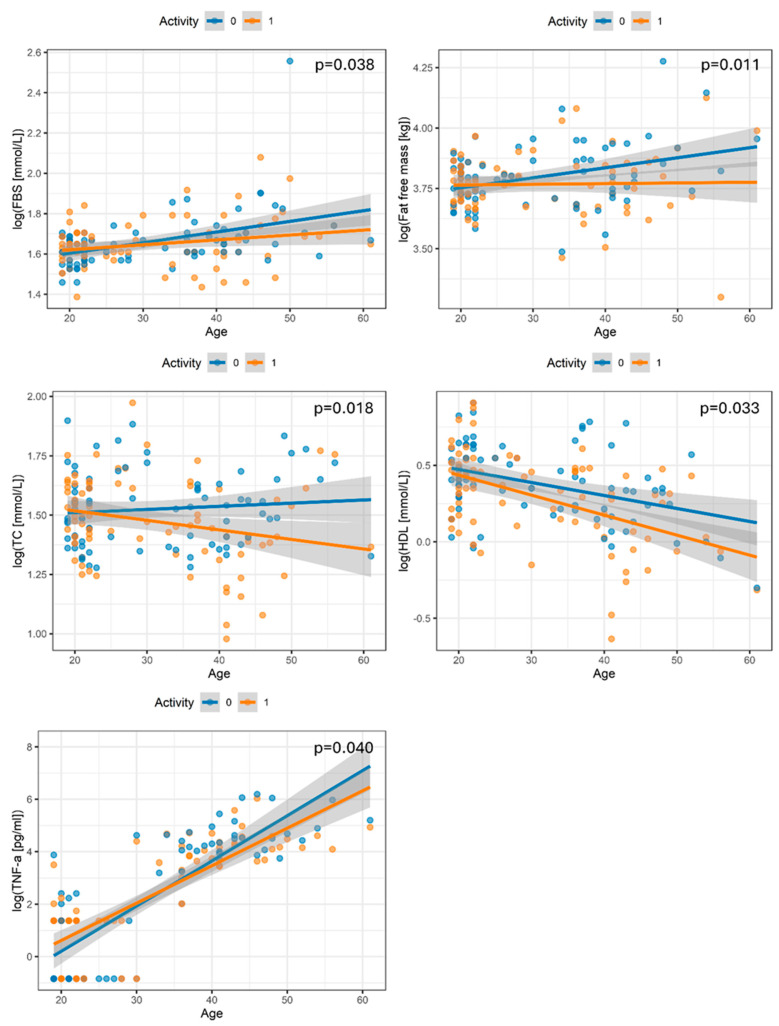
Age and physical activity jointly shape metabolic and physiological traits. Scatterplots show the relationship between age and each measured outcome before (blue) and after (orange) activity. Lines represent fitted linear regressions with 95% confidence intervals from mixed-effects model. The *p*-values indicate the significance of the interaction, testing whether the age-related slopes differ between before and after activity. Each point represents an individual measurement, and repeated measurements per participant are accounted for using subject-specific random intercepts.

Mixed-effects linear models were fitted to examine whether the association between physical activity and measured traits varied as a function of age, accounting for repeated measures within individuals and adjusting for BMI, fasting status, and activity duration. In the pre-training group, increasing age was associated with steeper increases in FBS, fat-free mass, total cholesterol, HDL-C, and TNF-α, whereas these age-related slopes were consistently attenuated or reversed in the physically active state, yielding significant interactions between age and activity for all shown traits (*p* ≤ 0.040). Specifically, older active participants exhibited flatter age gradients for FBS and fat-free mass, more pronounced declines in total and HDL cholesterol with age, amd a blunted age-related rise in TNF-α, indicating that regular physical activity modifies how age relates to cardiometabolic and functional parameters across adulthood, as shown in Figure 1.

Table 2 summarizes the estimated coefficients for the combined effect of age and activity on measured traits. Increasing age in the post training was associated with steeper declines in fat-free mass, total cholesterol, HDL cholesterol, fasting blood sugar, and TNF α compared with the sedentary state, as indicated by negative coefficients for the age-by-activity term for these traits (β range −0.00259 to −0.03575; *p* = 0.011–0.040), implying greater activity-related reductions among older than younger participants. Refer to Supplementary Table S1 for all measured traits.

## 4. Discussion

This study examined how a structured, moderate-intensity aerobic exercise program can modify age-group-specific responses to the training of metabolic, inflammatory, and functional markers in women. Using a within-subject design with measurements obtained before and after exercise intervention, the study evaluated whether exercise-induced adaptations differ between younger and older women, thereby clarifying the extent to which physical activity can offset age-related metabolic and inflammatory deterioration.

The within-subject design of this study, in which the measurements reflected before the exercise intervention and after completing the program, minimizes confounding by baseline differences and strengthens causal inference regarding the effects of physical activity. BMI, body composition, glucose, insulin, most lipid parameters, inflammatory markers, and telomere indices were comparable between conditions, indicating that the post-intervention changes are unlikely to be explained by pre-existing group differences. Against this stable baseline, the intervention produced a coherent shift toward a more active physiological profile, characterized by higher superoxide dismutase levels, lower TNF-α, increased weekly METs, a modest reduction in HDL cholesterol, and a trend toward improved insulin sensitivity. Collectively, these findings suggest that even a relatively short exercise program can induce meaningful changes in oxidative, inflammatory, and metabolic profiles in healthy adults. The observed rise in superoxide dismutase and reduction in TNF-α, together with the trend toward lower HOMA-IR, indicates that enhanced redox balance and attenuated inflammatory signaling may underlie the metabolic benefits of the intervention, even if improvements in insulin sensitivity do not meet conventional thresholds for statistical significance [27,28]. Promoting regular physical activity across adulthood, even when it produces only modest, multidimensional improvements in glucose, lipids, inflammation, and functional capacity, may help shift individuals onto lower-risk aging trajectories rather than simply improving single biomarkers at a single time point.

Beyond simple pre–post contrasts, the mixed-effects models offer insight into how physical activity modifies age-group-specific responses to the training of key traits. Significant Age × Activity interactions for FBS, fat-free mass, total and HDL cholesterol, and TNF-α indicate that aging follows different slopes in post-training versus pre-training individuals. In older participants, physical activity was associated with more favorable age-related patterns, specifically lower FBS, lower total cholesterol, and a blunted age-related increase in TNF-α, suggesting the partial attenuation of age-related metabolic and inflammatory deterioration. These findings build on previous work by demonstrating that exercise not only shifts average levels of risk markers but also reshapes the trajectory of how these markers change with age.

Consistent with this interpretation, numerous studies, including systematic reviews and meta-analyses, have shown that both aerobic and resistance training effectively reduce circulating TNF-α level in older adults, reflecting improvements in systemic inflammation and immune function [29]. Exercise exerts its anti-inflammatory effects through multiple mechanisms, inducing anti-inflammatory myokines such as exercise-stimulated IL-6 and IL-10, inhibiting TNF-α production and enhancing metabolic and vascular health, all of which further inhibit pro-inflammatory signaling [30]. Collectively, these results and supporting literature show that regular physical activity can shift the cytokine environment toward a more anti-inflammatory profile in later life [29,31], aligning with current concepts of “inflammaging,” in which chronic low-grade inflammation and oxidative stress are key drivers of cardiometabolic risk.

Regular exercise significantly attenuates age-related increases in FBS in older women, who are more prone to elevated FBS with age, whereas younger females typically maintain lower baseline levels. These glycemic benefits are achieved by improving insulin sensitivity, enhancing muscle glucose uptake primarily via increased GLUT4-mediated glucose transport in skeletal muscle [32]. Collectively, these adaptations result in better glucose control and lowered metabolic risk in physically active women across midlife [33,34]. Recent meta-analyses confirm that combined aerobic and resistance exercise training improves glucose metabolism markers across age groups, with individuals at higher metabolic risk, such as older adults or those with elevated baseline FBS, experiencing the largest proportional improvements [7,35]. A recent 2024 tracer-based investigation confirmed that age significantly alters glucose kinetics: older healthy adults exhibited reduced glucose disposal and increased gluconeogenesis after a glucose challenge, although fasting values were not dramatically different from younger subjects in all populations. This aligns with longstanding evidence of age-associated impairment in insulin-mediated glucose disposal [36]. A 2024 meta-analysis of 24 randomized controlled trials found that combined exercise interventions significantly reduced FBS in non-diabetic sedentary adults, with the most pronounced benefits seen in participants starting with higher FBS, a group that tends to be older [7]. Additionally, an early 2025 trial of structured physical activity, with or without dietary changes, reported marked reductions in FBS (from 10.0 to 6.3 mmol/L) among predominantly middle-aged adults over six months, demonstrating that significant metabolic improvements remain achievable in midlife and beyond [37].

On the other hand, physical activity has the greatest positive impact on total cholesterol levels in older women compared to younger, showing a strong negative age × activity interaction. Active older women experience more significant reductions in total cholesterol than less active ones. However, HDL declines more steeply with age in both active and inactive older women. Regular exercise appears particularly beneficial for lipid management in older women, as they show the most marked improvements in total cholesterol. Consistent evidence indicates that exercise lowers total and LDL cholesterol, especially among postmenopausal and older women [38]. Recent studies consistently show that engaging in regular physical activity, especially aerobic and resistance training, leads to significant reductions in total cholesterol among older women. In meta-analyses and intervention trials, improvements in lipid profiles are more pronounced in active older women, with reductions in total cholesterol ranging from 8% to 12% after sustained exercise programs. These patterns support the interpretation that physical activity attenuates age-related dyslipidemia, with stronger effects observed among older women compared to younger cohorts [39]. In the large Chinese cohort studied by Feng et al. [40], total cholesterol and LDL-C in women increase with age until about 60, after which they stabilize or decline, while HDL-C exhibits irregular patterns but is generally higher than in men until late life, at which point it may begin to decrease. Together, these findings show that although aging and menopause can bring about increases in cholesterol levels, especially total and LDL cholesterol, the nature and function of HDL also change in ways that may increase cardiovascular risk for older women [40]. A 12-week physical exercise program has been shown to improve overall lipid profiles, but the extent of benefit is influenced by age and baseline physiological factors. While younger or more adaptable individuals may see greater changes, sustained physical activity remains a central strategy for managing dyslipidemia in adult women [41]. Active older women show significant improvements in total cholesterol with regular physical activity. HDL-C declines with age and is less responsive to exercise, especially in postmenopausal women. These trends highlight the need for targeted prevention and routine lipid screening in older women [41]. The Study of Women’s Health Across the Nation (SWAN) tracked midlife women over a 17-year period and identified distinct patterns in both physical activity and HDL cholesterol levels. The largest group had consistently low physical activity and low HDL levels. Surprisingly, consistently high activity was associated with moderate, but not high, HDL levels. Overall, no clear or direct association was observed between physical activity patterns and HDL levels in this large cohort [42]. Some longitudinal cohort studies, including the Medical Research Council National Survey of Health and Development, indicate that although HDL levels generally decline with age, physical activity can help slow this decline. However, the observed effect is typically modest and less pronounced than improvements seen for other lipid fractions [42,43]. Recent reviews also suggest that in older women, HDL cholesterol responses to interventions may be limited due to the combined influences of aging, menopause, and inflammation-related changes in HDL metabolism [44].

Younger women demonstrated better preservation of fat-free mass following exercise, whereas older women exhibited lower response, with less gain or even a decrease in Fat-Free Mass (FFM). This age-related difference likely reflects underlying anabolic resistance where aging skeletal muscle becomes less responsive to exercise-induced hypertrophy and protein synthesis [45]. Key contributing mechanisms may include the diminished activation of molecular signaling pathways (such as mTOR), impaired amino acid delivery, and hormonal changes (notably menopause-related estrogen and IGF-1 decline) that reduce muscular adaptability [46,47,48]. The aging process is also associated with sarcopenia, the loss of satellite cell function, increased chronic inflammation (e.g., TNF-α, IL-6 elevation), mitochondrial dysfunction, and reduced vascular health, all further impeding muscle growth and repair after exercise. Additionally, lower habitual physical activity and dietary protein intake compound anabolic impairment in older women [49].

Despite these limitations, appropriately designed exercise interventions can still provide significant benefits, including improvements in muscle strength, metabolic health, slowed fat mass accumulation, and the preservation of physical function [50]. To optimize muscle adaptations, older women may require tailored resistance training regimes alongside higher protein diets and strategies addressing inflammation and vascular health [51]. Further contributing to age-related muscle loss is the increased activation of the NF-κB pathway (which promotes muscle atrophy), chronic oxidative stress, and vascular impairment that restricts nutrient and oxygen delivery to muscle tissue. Collectively, these factors reduce muscle protein synthesis, impair regeneration, and ultimately result in a blunted anabolic response in older women, even when exercise and nutritional interventions are in place [48]. These findings underscore the need for multimodal approaches to preserve or enhance FFM in aging females, a critical aspect of maintaining physical function and metabolic health into later life.

On the other hand, no significant age-by-physical activity interaction was observed for other cytokines, including IL-1β, IL-1RA, IL-6, IL-8, IL-10, and MCP-1, as well as for oxidative stress markers (catalase and SOD). Similarly, total weekly physical activity (measured by METs), anthropometric measures (weight, BMI, body fat percent, fat mass, muscle mass, triglycerides), and telomere length did not demonstrate significant age interaction effects with physical activity in this study.

This result suggests that the effect of physical activity on these parameters is broadly similar across different age groups of women in this cohort and their combined effect may not always result in synergistic changes, especially when confounding lifestyle or genetic factors exist [52]. Importantly, the absence of significant interactions in telomere length aligns with several recent reviews showing that habitual physical activity can help maintain telomere length across adulthood, but may not induce strong age-specific effects unless exercise intensity or duration is high [53,54]. Similarly, oxidative stress markers and certain cytokines might require longer-term interventions, different exercise modalities, or additional nutritional support to reveal meaningful age differences [54].

## 5. Conclusions

In conclusion, a relatively short moderate-intensity aerobic training program in women was sufficient to improve antioxidant defenses, lower inflammatory status, and result in favorable trends in insulin sensitivity, despite minimal changes in body composition and telomere length. Age-by-activity interactions further indicated that physical activity can reshape age-group-specific responses to training in glycemic control, lipids, inflammatory markers, and functional capacity, partly mitigating adverse age-associated changes, particularly among older participants. Collectively, these findings support promoting regular physical activity throughout adulthood as a means to modify the course of metabolic and inflammatory aging, while emphasizing the need for longer-term studies to determine whether these physiological benefits translate into reductions in clinical risk.

### Study Limitation

We acknowledge several limitations that warrant consideration. These include the small sample size, absence of a non-exercising control group, and the confounding of age with training duration/dose. Although we adjusted for training duration in our analyses, future studies with harmonized exercise protocols and control groups are needed to disentangle age-specific effects from training dose. Additionally, despite statistical adjustment for fasting status, residual confounding from Ramadan fasting and later session timing cannot be fully excluded; results for fasting-sensitive metabolic variables (e.g., glucose, insulin, triglycerides) should therefore be interpreted cautiously.

## Figures and Tables

**Table 1:** Baseline characteristics of the participants in this study, grouped into pre and post training.

	Pre Training (*n* = 79)	Post Training (*n* = 79)	*p*-Value
Exercise Duration			
8 weeks	50	50	0.999
4 weeks	29	29	
Age	28 (21–41)	28 (21–41)	0.982
BMI	27.66 (5.89)	27.25 (5.39)	0.793
HOMA-IR	2.68 (1.64–3.95)	2.21 (1.54–3.72)	0.072
FBS (mmol/L)	5.2 (4.9–5.45)	5.1 (4.9–5.5)	0.437
Insulin m U L	10.52 (6.66–16.77)	10.06 (6.98–15)	0.327
Catalase μ mL	20.05 (19.1–20.39)	20.18 (19.47–21.16)	0.078
SOD μ mL	0.76 (0.69–1.01)	0.94 (0.78–1.12)	0.001
IL 1BETA (pg/mL)	0.47 (0.2–30.85)	0.78 (0.47–24.44)	0.304
IL 1RA (pg/mL)	115.66 (29.28–312.48)	103.74 (20.56–381.71)	0.640
IL 6 (pg/mL)	11.64 (7.68–24.77)	16.1 (7.68–24.77)	0.859
IL 8 (pg/mL)	2.95 (0.87–19.78)	5.86 (1.23–17.93)	0.717
IL 10 (pg/mL)	0.68 (0.23–14.09)	0.69 (0.23–13.15)	0.050
MCP-1 (pg/mL)	421.59 (227.05–651.99)	488.6 (298.2–649.36)	0.767
TNF-α (pg/mL)	11.18 (0.43–83.02)	6.64 (3.95–61.12)	0.002
TC (mmol/L)	4.6 (4–5.11)	4.36 (3.94–5.02)	0.074
TG (mmol/L)	0.75 (0.59–0.98)	0.75 (0.56–1.04)	0.474
HDL (mmol/L)	1.51 (0.38)	1.39 (0.39)	0.019
LDL (mmol/L)	2.69 (2.37–3.36)	2.71 (2.29–3.06)	0.320
Relative telomere length T S ratio	0.36 (0.29–0.57)	0.37 (0.26–0.58)	0.709
average telomere length on each chromosome end	2.94 (2.28–4.56)	3.28 (2.21–4.74)	0.231
Total MET value per week	1155 (670.5–2132)	1794 (1084.12–3152)	0.004
Weight (kg)	68.8 (61.4–79.9)	67.65 (60.6–78.65)	0.555
Body fat	36.3 (31.4–40.6)	36.4 (31.4–40.75)	0.287
Fat-free mass (kg)	44.4 (41.05-47.7)	43.2 (39.9–46.6)	0.458
Fat mass (kg)	25.4 (18.4–32.8)	25.8 (18.65–32.5)	0.339
Muscle mass (kg)	40.5 (37.7–44.3)	39.8 (35.15–43.15)	0.108

Data are expressed as mean (standard deviation) or median (interquartile range) after assessing normality using the Shapiro–Wilk test. Comparisons between the pre- and post-training groups were conducted using either paired Student’s T-test or the paired Wilcoxon signed rank test, as appropriate. A *p*-value < 0.05 was considered statistically significant.

**Table 2:** Mixed effects model estimates for the combined effect of age and physical activity on metabolic and physiological traits.

Metabolite	β	SE	*p* Value
Fat-free mass (Kg)	−0.00259	0.0009	1.07 × 10^−2^
TC mmol/L	−0.00528	0.0022	1.84 × 10^−2^
HDL mmol/L	−0.00582	0.0027	3.30 × 10^−2^
FBS mmol/L	−0.00273	0.0013	3.84 × 10^−2^
TNF-α pg/mL	−0.03575	0.0171	4.00 × 10^−2^

Β coefficients represent the age-by-activity term from the linear mixed-effects models. Negative β values indicate steeper age-related declines in the post-training state compared with pre-training state. Models were adjusted for BMI, fasting status, and activity duration, with participants included as a random effect. A *p*-value of <0.05 was considered statistically significant.

## Data Availability

Data are available from the corresponding author upon reasonable request.

## Supplementary Materials

The following supporting information can be downloaded at: https://www.mdpi.com/article/10.3390/biomedicines14030575/s1, Table S1: Supplementary to Table 2, summarizing the estimated coefficients for the combined effect of age and activity on the measured traits.
